# Effect of Spectral Quality of Monochromatic LED Lights on the Growth of Artichoke Seedlings

**DOI:** 10.3389/fpls.2017.00190

**Published:** 2017-02-17

**Authors:** Roel C. Rabara, Glenn Behrman, Thomas Timbol, Paul J. Rushton

**Affiliations:** ^1^Texas A&M AgriLife Research and Extension CenterDallas, TX, USA; ^2^CEA Advisors LLCDallas, TX, USA

**Keywords:** light-emitting diodes, LED, spectral quality, photosynthetic photon flux density, artichokes, indoor farming, urban agriculture

## Abstract

Indoor farming is becoming a popular alternative approach in food production to meet the demand of a growing world population. Under this production system, artificial light provides the main source of illumination in sustaining plant growth and development. The use of light-emitting diodes (LEDs) is a popular source of artificial light for indoor farms due to its narrow light spectra, modular design and energy efficiency. This study purposely assessed the effect of monochromatic LED light quality on the growth of three varieties of artichoke seedlings compared to greenhouse condition. Spectral quality assessment showed that photosynthetic photon flux density (PPFD) was highest under red LED light, but only a third of the total PPFD under natural light. Seedlings grown under red light showed 60–100% more shoot dry weight and were 67–115% taller than seedlings grown in the greenhouse. However, seedlings under blue or white light conditions showed 67–76% less in biomass compared to greenhouse-grown seedlings. Overall, plant response of seedlings under red light condition was much better compared to greenhouse-grown seedlings emphasizing the importance of red light spectral quality in plant growth and development.

## Introduction

The world population is estimated to reach the nine-billion mark by 2050 of which 66% will be living in urban areas (UN, 2014). This growth in population means that a 70–100% increase in food production is needed to feed the growing world population (Godfray et al., 2010). One option to increase food production is through urban agriculture. This type of agriculture could also take the pressure off rural agriculture and decompensate land loss (Eigenbrod and Gruda, 2015). In developing countries, urban agriculture supplies up to 90% of vegetables consumed in the cities and about 100 million urban farmers worldwide provide cities with fresh horticultural goods (Orsini et al., 2013).

Indoor farming has become a popular form of urban agriculture as an alternative approach in food production. Artificial light source is a critical component in indoor farming since light is one of the most important environmental factors affecting plant growth and morphology (Hernández and Kubota, 2016). Fluorescent tubes (FTs) and high intensity discharge (HID) lamps (e.g., high pressure sodium) are the most commonly used artificial light sources for plant research and greenhouse horticulture (Hogewoning et al., 2010). However, FTs lack the sustained photosynthetic photon flux (PPF) capability necessary for high crop productivity (Massa et al., 2007). Rapid advances in lighting technology now provide several supplemental lighting options for indoor farming. Light-emitting diodes (LEDs) denote a fundamentally different technology having advantages over traditional lighting systems currently used in greenhouses. LEDs are durable, have long lifetimes, high radiant efficiency, and relatively narrow emission spectra (Massa et al., 2008; Morrow, 2008). Horticultural LEDs also provide options to select specific wavelengths for a targeted plant response. This inherent advantage of LED lights makes them an important light source for plant growth, however research on the effect of spectral quality on plant growth and development is limited (Hernández and Kubota, 2016).

Light quality has specific effects on various plant responses such as photosynthesis, phototropism, photomorphogenesis, and photonasty (Hogewoning et al., 2010). Several studies have shown the effect of spectral quality on plants' photosynthetic activities *in planta* and *in vitro* (Lee et al., 2007; Wang et al., 2016). Rate of photosynthesis in *Withania somnifera* plantlets increased with the increase of photon flux density up to 60 μmol m^−2^s^−1^ (Lee et al., 2007). In lettuce, photosynthetic rate increased with a decrease in the red to blue (R/B) ratio until 1 (Wang et al., 2016).

In this study, three different monochromatic LED lights were utilized to expound on the effect of light quality on the growth of artichoke seedlings. Artichoke, primarily produced in California is one of the important vegetable crops in the US valued at $ 73 M (NASS, 2016). Over half of artichokes produced in California are grown as perennials but growing artichokes as annuals is gaining interest since its production can be timed to mature at different times of the year to fill market niches (Smith et al., 2008). Artichokes grown as annuals are established as seedlings in greenhouses and then later transplanted into farm fields. Some advantages of growing artichokes as transplants are avoidance of weeds and diseases problems (Smith et al., 2008) and transplantation in areas with colder climates (Welbaum, 1994). Annual planting of artichokes requires vernalization to initiate the production of edible flower buds (Rangarajan et al., 2000). This cold treatment can be done easily in indoor farming facilities which make these facilities highly suitable for artichoke transplants production. Enhancing the growth of seedlings in indoor farms will support the interest of annual planting of artichokes for off-season production. Hence, this study aimed to identify what monochromatic LED lights most influence the growth of artichoke seedlings for transplants production under indoor farming facilities.

## Materials and methods

### Plant material propagation and light treatment

Seeds of artichoke (var. “Green Globe,” “Cardoon,” and “Violetto”; Territorial Seed Co., Cottage Grove, OR, USA) were germinated in 72-cell plug trays in the greenhouse. At two-leaf stage, artichoke seedlings were transplanted into cone-tainers (3.8 × 21 cm) (Stuewe & Sons, Tangent, OR, USA) with SunGro soil medium (SunGro, Agawam, MA, USA). Then the seedlings were transferred to a growth room and were placed on light shelves with individual light-emitting diode (LED) LumiGrow Pro 325 light sources (LumiGrow, Emeryville, CA, USA) set at red, white and blue lighting conditions. Another set of seedlings was left in the greenhouse for comparison. Black cloth was used to cover each shelf to prevent light contamination. Plants were grown in a 16/8 h photoperiod (day/night) at 22°C temperature. Spectral quality for each light condition (Table 1) was measured using a Lighting Passport spectrometer (AsenseTek, Taipei, Taiwan) interfaced with Android mobile phone applications.

**Table 1:** **Spectral output of the different light sources used in growing artichoke seedlings**.

**Parameters**	**Light condition**			
	**Natural**	**Red**	**White**	**Blue**
**TOTAL PPFD (μmol m^−2^s^−1^)**				
PPFD (400–700 nm)	788.84	236.54	21.44	41.14
PPFD Infrared (701–780 nm)	220.03	1.86	1.71	0.11
PPFD Red (600–700 nm)	293.07	233.06	9.93	0.11
PPFD Green (500–599 nm)	285.55	0.48	8.87	0.24
PPFD Blue (400–499 nm)	210.19	3.02	2.65	40.79
PPFD UltraViolet (380–399 nm)	18.207	0.05	0.02	0.06
YPFD (380–780 nm)	701.03	216.54	19.41	30.56
YPFD IR (701–780 nm)	31.264	0.53	0.36	0.01
YPFD R (600–700 nm)	262.82	213.34	9.25	0.10
YPFD G (500–599 nm)	245.17	0.44	7.86	0.20
YPFD B (400–499 nm)	150.99	2.23	1.93	30.21
YPFD UV (380–399 nm)	10.732	0.03	0.01	0.04
Red/Blue ratio	1.3943	77.22	3.75	0.00
Red/Infrared ratio	1.332	125.22	5.79	1.01
Daily light integral (mol m^−2^d^−1^)	68.156	20.44	1.85	3.55
Illuminance (Lux)	44679	2120.30	1429.20	334.71
Peak wavelength (λp)	479	666.00	603.00	447.00
Dominant wavelength (λD)	548.9	0.00	582.58	452.29
CCT	5530	0.00	3166.60	0.00
CRI(Ra)	97.559	0.00	83.63	0.00
Distance of light source from plants (cm)	–	82.55	82.55	82.55

### Phenotyping, biomass, shoot/root ratio measurements

After a month under varying light conditions, the artichoke seedlings' plant height, root length, biomass and leaf number were measured. Plant height was measured from the base of the plant to the tip of the longest leaf. Total number of leaves include leaflets observed for each genotype. Shoot biomass was harvested by cutting the shoot at the base while root biomass was determined after carefully removing soil from the roots using a sieve to minimize root loss. Both tissues were dried in an oven set at 60°C for 48 h and then dry weights were obtained.

### Chlorophyll content quantification

Leaves were harvested from each genotype and were flash frozen in liquid nitrogen then stored in a −80°C freezer until further processing. Tissues were ground to fine powder and total chlorophyll was extracted by 80% acetone in the dark. Extraction was performed following the protocol outlined by Ni et al. (2009). Absorbance was measured using the UV/Vis option in a Thermo Scientific Nanodrop 2000c spectrophotometer (Thermo Scientific, Waltham, MA, USA) at 645 and 663 nm wavelength. Chlorophyll a, b, and a+b were calculated using the methods described by Ni et al. (2009).

### Digital image analysis of leaf color

Digital image analysis of leaf color was done using the second fully expanded leaf harvested from each genotype. A total of five plants per genotype were sampled. Digital images were taken using a Canon EOS Rebel T2i digital single-lens reflex (dSLR) camera (Canon, Melville, NY, USA) at a shutter speed of 1/60 s, f-stop set at f/5.6, and focal length of 55 mm. The camera was mounted on a tripod and fixed at a distance of 51 cm from the leaf samples. The LED light source was placed at the same distance from the plants. The digital images were saved in JPEG format at a size of 5,184 × 3,456 pixels and were downloaded to a personal computer for analysis using ImageJ software. Batch measurement of the RGB values from each digital image was conducted using a macro developed for ImageJ software. The RGB values were converted to HSB (hue, saturation, brightness) values using the colorsys module in Python. The Dark Green Color Index (DGCI) was calculated using the formula: DGCI = [(H − 60)/60 + (1 − S) + (1 − B)]/3 (Karcher and Richardson, 2003). The calculations were programmed in MS Excel spreadsheet to automate the value conversion process.

### Statistical analysis

The experiment was designed in a two-factorial complete randomized design with five replications per treatment. Five plants per light treatment was used to ensure that each plant received similar light intensity and quality within the shelf. One-way analyses of variances (ANOVA) were used for significance tests of the treatment effects. *Post hoc* Tukey's honest significant difference (HSD) tests were done on significantly different treatment means. All statistical analyses were done using JMP statistical software (SAS, Cary, NC, USA). Normality of residuals was assessed by normal quantile plot and tested for goodness-of-fit using Shapiro-Wilk *W*-Test. Tests of homoscedascity of variances were done using four different tests (O'Brien, Brown-Forsythe, Levene, and Bartlett) for each factor (light treatment, variety, and light × variety interaction; Supplemental Table 3).

## Results

### Spectral quality of different growing light treatments

**Figure 1:**
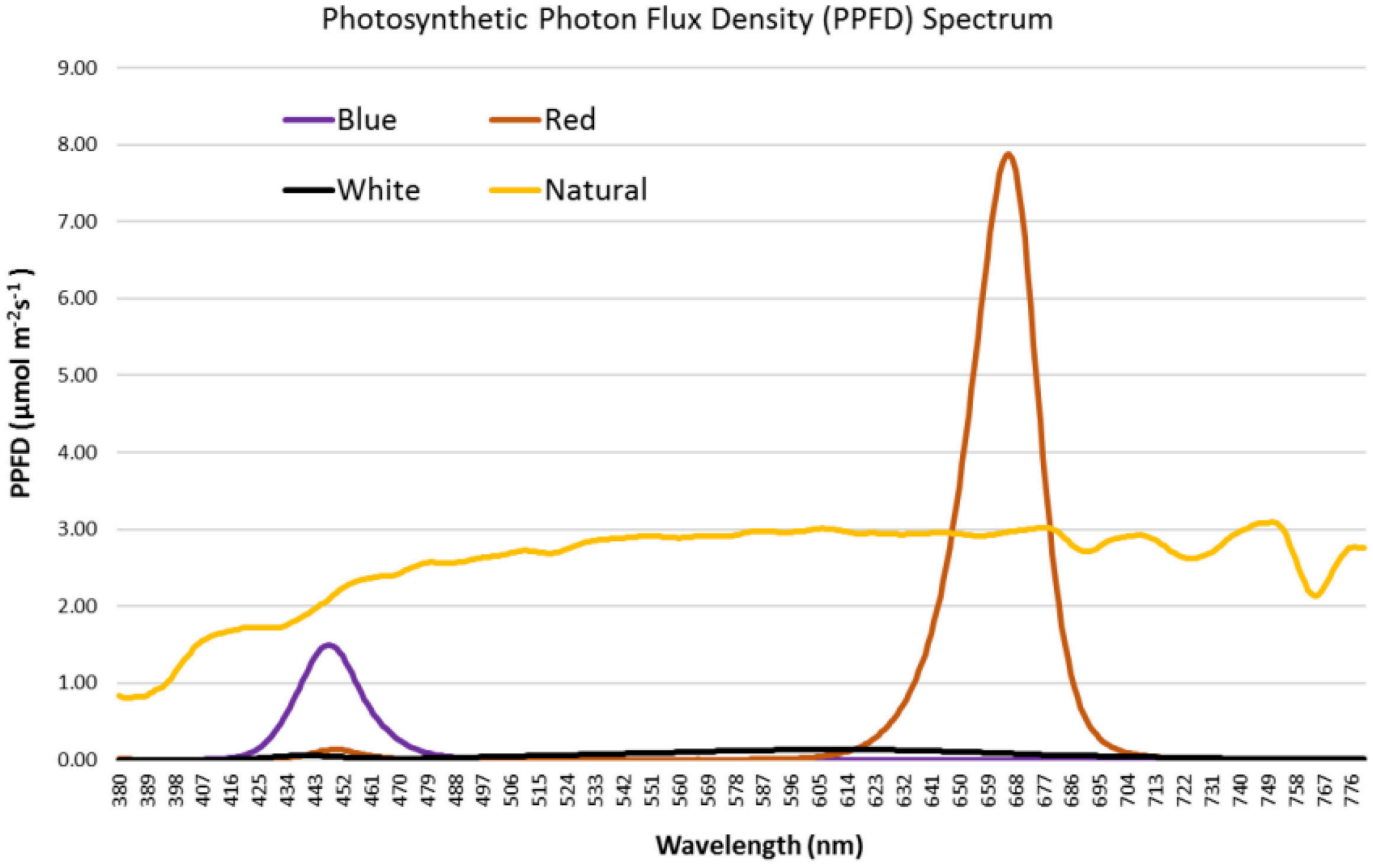
**PPFD (μmol m^**−2**^s^**−1**^) spectrum of natural light and red, white and blue LED lights used in growing artichoke seedlings**.

Monochromatic LED lights provide specific light spectrum that can be used to assess the effect of precise spectral quality on the growth and development of crops. In our study, spectral quality for each light treatment was measured and Figure 1 shows the photosynthetic photon flux density (PPFD) spectrum for each light treatment. Clearly, the light treatments used to grow the artichoke seedlings have a diverse PPFD spectrum. The PPFD spectrum under natural light was broad and the highest value observed (3.089 μmol m^−2^s^−1^) was at 750 nm. For the blue LED light, the spectrum was narrow (423–478 nm) with the highest PPFD value (1.497 μmol m^−2^s^−1^) observed at 448 nm. For the red LED light, the spectrum was also narrow (623–695 nm) with highest PPFD value (7.879 μmol m^−2^s^−1^) observed at 666 nm. In contrast, white LED light showed low PPFD values ranging between 0 and 0.135 μmol m^−2^s^−1^ and the maximum value observed at 603 nm. Table 1 sums up the spectral quality of the varied light treatments used in this study. The natural light treatment provided the highest total PPFD (788.84 μmol m^−2^s^−1^) in the region of 400–700 nm wavelength. Red light treatment came in second at 236.54 μmol m^−2^s^−1^, while white light had the lowest total PPFD (21.44 μmol m^−2^s^−1^) among all light treatments. Among the different light treatments, R/B ratio was highest in red light (77.22) followed by white and natural lights, at 3.75 and 1.39, respectively. Illuminance was high under natural light at 44,679 lux followed by red, white and blue light at 2,120, 1,429, and 335 lux, respectively.

### Phenotypic differences among genotypes under varying light conditions

**Figure 2:**
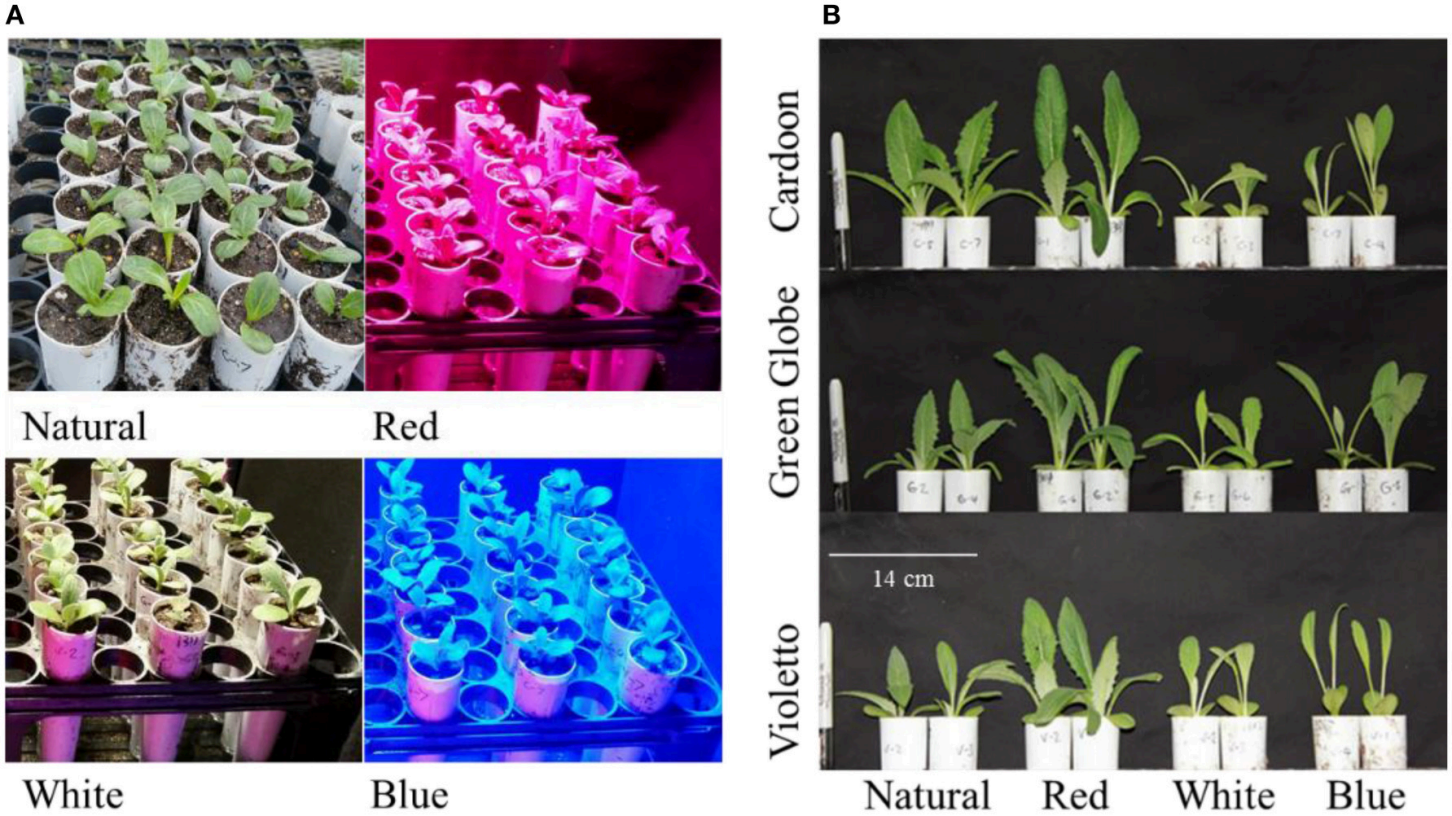
**Artichoke seedlings grown under various light conditions (A)** and phenotypic differences observed after two weeks **(B)**.

**Figure 3:**
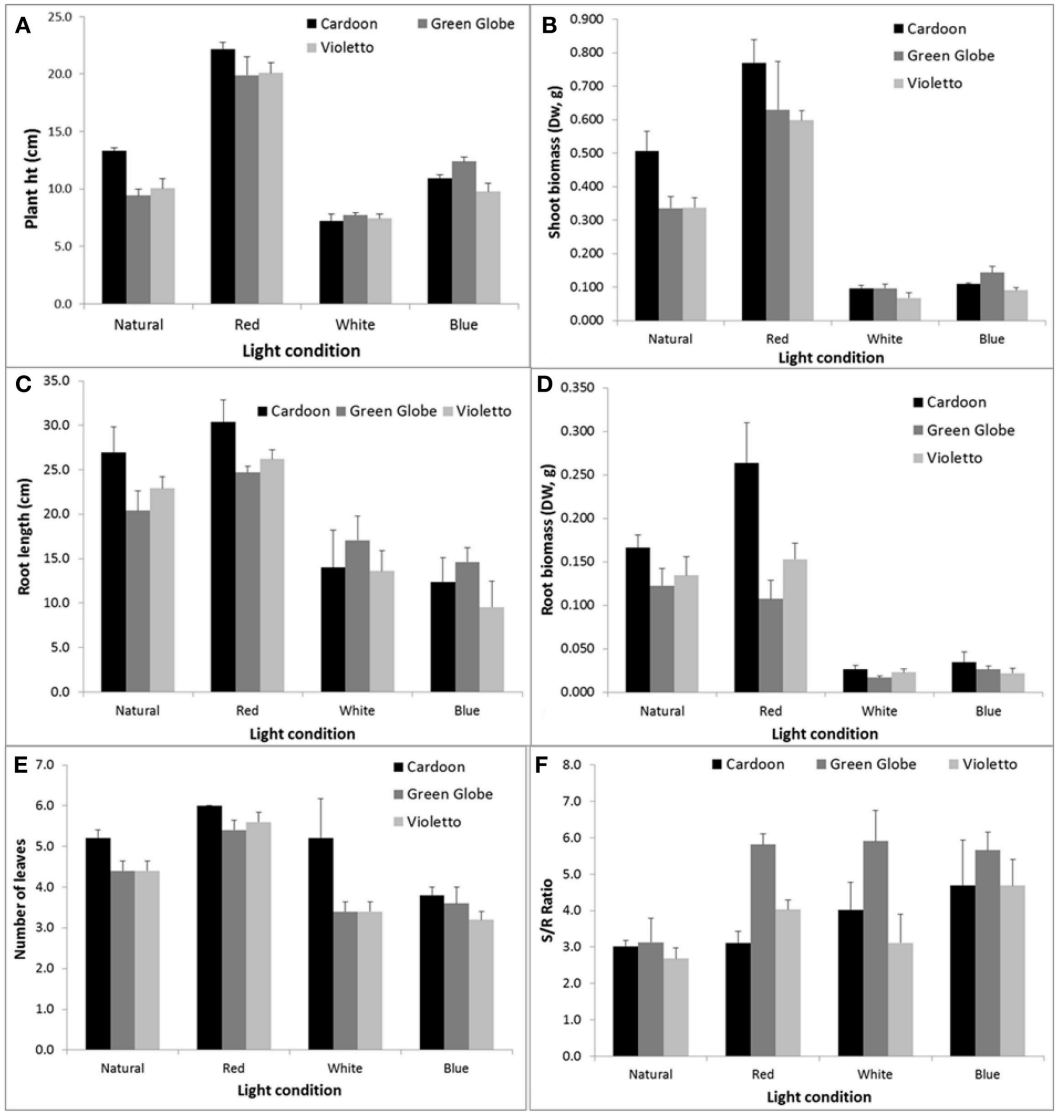
**Plant height (A**,cm), root length (**C**, cm) and shoot (**B**, g), root biomass (**D**, g), number of leaves **(E)** and shoot/root ratio **(F)** of artichoke grown under varying light conditions. Error bars represent the SEM.

The effects of narrow band light spectrum on the growth of three artichoke varieties were assessed using monochromatic LED lights. The spectral quality of these LED lights significantly influenced the growth of artichoke seedlings (Figure 2). The influences of light treatments on the growth and development of seedlings became apparent just after two weeks as shown in Figure 2B. Seedlings under red light showed doubling in plant height across all artichoke genotypes compared to greenhouse-grown seedlings under natural light conditions. A contrasting effect was observed in artichoke seedlings under white light when compared to seedlings under natural light conditions. Under white light, seedlings were shorter in height (0.5–0.8x) compared to greenhouse-grown seedlings. No significant difference in plant height was observed in seedlings grown under blue and natural light treatments. The comparisons of plant characters of artichokes grown under monochromatic light treatments against natural light condition were summarized in Supplemental Figure 1. Statistical analyses showed that plant variety, light treatment, and interaction had a significant influence on plant height (Supplemental Table 1). Varietal differences (Cardoon and Violetto) have significant influence on plant height (*p* = 0.0156). Influence of light treatment on plant height was highly significant (*p* < 0.0001). Under red light treatment, artichoke seedlings were taller (average of 22 cm) compared to plants under blue (11 cm), natural (11 cm), and white (7 cm) light conditions. The interaction between light treatment and artichoke genotype was apparent in all genotypes under red light (Supplemental Table 2). Under red light, Cardoon was the tallest (22 cm) compared to Violetto (20 cm) and Green Globe (20 cm) (Figure 3). Cardoon variety grown under white light was the shortest (7 cm) among other genotypes under various light treatments. On the average, seedlings grown under white light were 64% shorter than the greenhouse-grown seedlings (Supplemental Figure 2).

In contrast, root length was not influenced by genotype but only by light treatment (*p* < 0.0001). Root lengths of plants under red (27 cm) and natural (23 cm) light conditions were the longest and showed similar influence across all genotypes tested (Figure 3C). Artichokes subjected to blue and white light conditions had the shortest root length of 12 and 15 cm, respectively (*p* = 0.05).

Light quality also affected leaf morphogenesis. This study showed that leaf development was influenced by both genotype and light treatment (Figure 3E). The Cardoon variety developed more leaves than the Green Globe and Violetto artichoke seedlings. Overall, artichokes seedlings grown under the red light produced more leaves compared to seedlings grown under natural, white, and blue light conditions. Also, leaves of seedlings under the red light were thicker than the leaves of other seedlings under natural, white, and blue light treatments. Above- (shoot) and below-ground (root) biomass showed significant changes under various light treatments (Figures 3B,D). Plant shoot biomass was influenced by both genotype (*p* = 0.0356) and light conditions (*p* < 0.0001) (Supplemental Table 1). Similarly, root biomass was also influenced by genotype (*p* = 0.0004) and light conditions (*p* < 0.0001), and the interaction of the two factors (*p* = 0.0047) as well. Genotype had a significant influence on biomass as shown by the Cardoon variety which was significantly different from Violetto (*p* = 0.05). Light spectral quality has significant influence on artichoke genotype biomass. Statistical analyses showed that Cardoon was significantly different from Violetto (*p* = 0.05), but not significantly different from Green Globe. Further, seedlings' shoot biomass was least for artichokes grown under white light (0.097 g) and significantly higher (8-fold) for those grown under red light. The blue light-treated seedlings resulted in a 6-fold decrease in the biomass compared to those exposed to red light. The same trend was noted in the root biomass for seedlings that underwent white and blue light treatments. Calculations of shoot/root ratio showed that Green Globe had the highest value (5.1) compared to Cardoon (3.7) and Violetto (3.6). Green Globe had the highest S/R ratio because it produced less root biomass compared to the two other varieties. Overall, seedlings grown under red light condition resulted in 22–97% increase in growth compared to natural light condition (Supplemental Figure 2).

### Effect of light on chlorophyll content and greenness of artichoke seedlings

**Figure 4:**
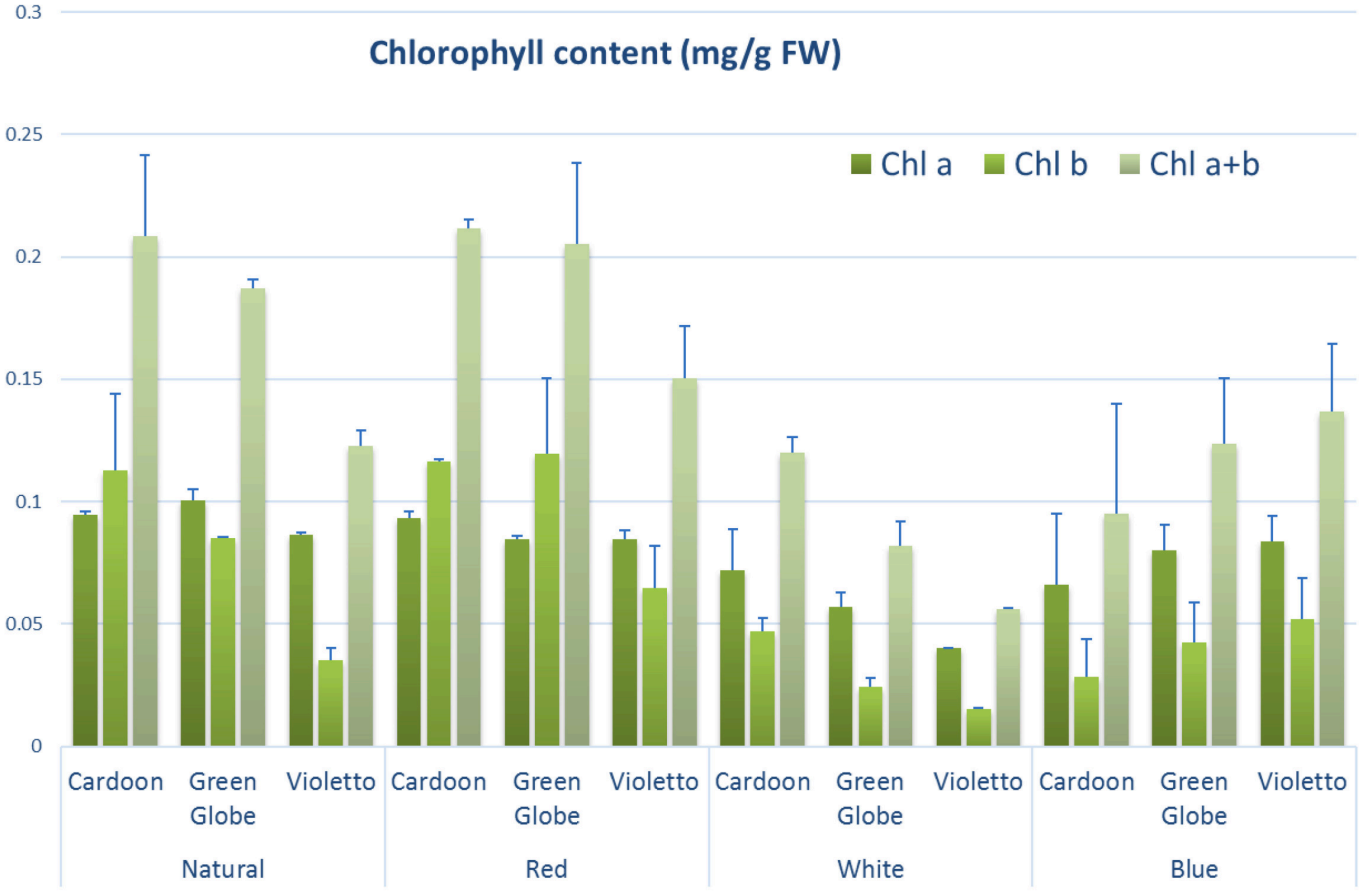
**Chlorophyll concentration (mg/g FW) of different artichoke varieties grown under varying light conditions**. Bars indicate SE of the means using five biological replicates.

Chlorophyll content measurement is commonly used to assess plant growth and vigor (Ni et al., 2009) as its concentration is highly correlated with the rate of photosynthesis (Emerson, 1929). Like other morphological characters measured in this study, chlorophyll content was significantly affected by light treatment (*p* = 0.0035) (Figure 4). Accumulation of chlorophyll did not vary among artichoke varieties. Chlorophyll content of seedlings under natural, red and blue light treatments were also not significantly different. However, total chlorophyll of plants under red light was higher than those grown under natural light (Supplemental Table 1), but was not statistically significant.

**Figure 5:**
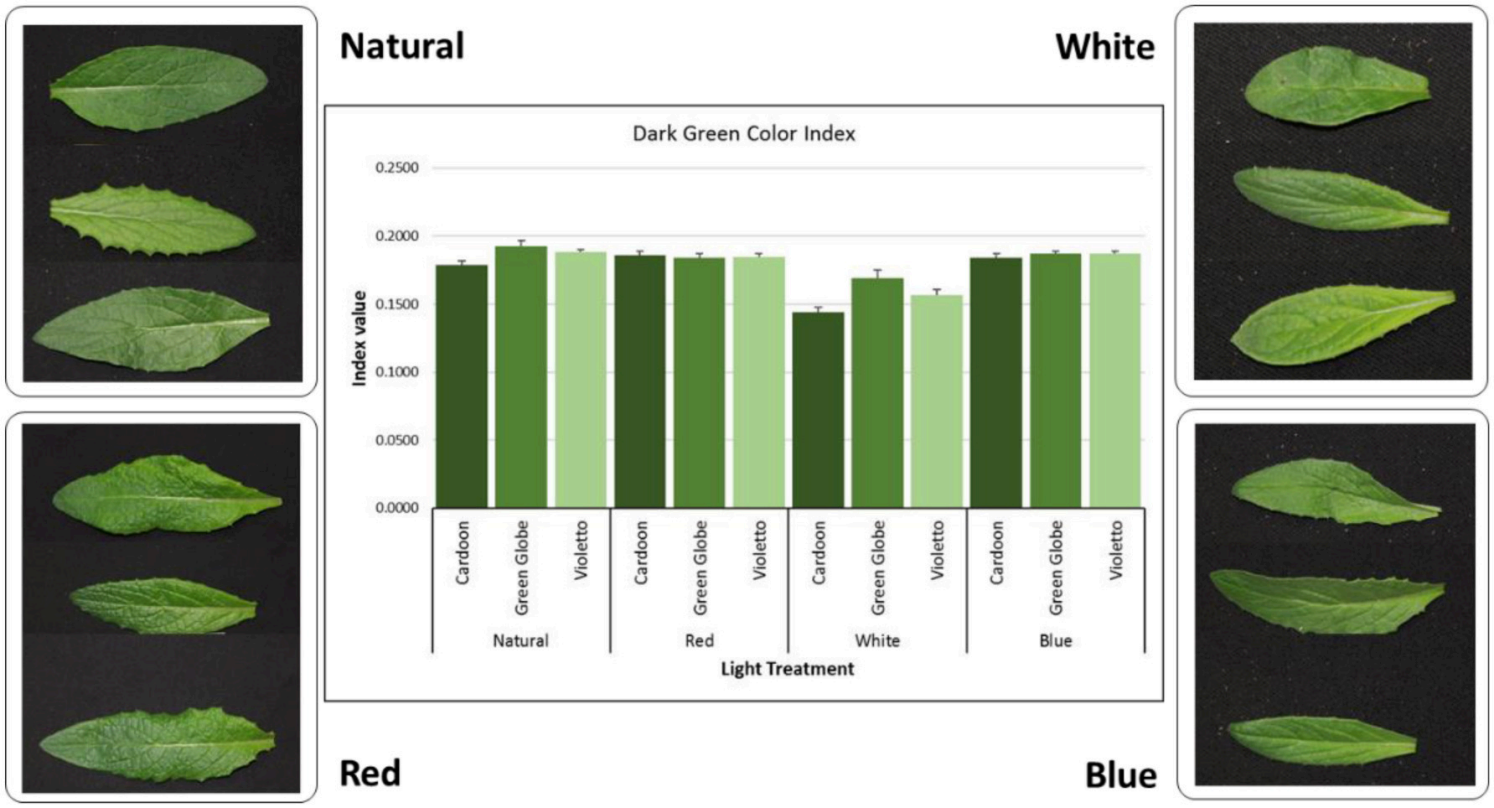
**Digital image analysis (DIA) of green color in artichoke leaves**. Dark green color index (DGCI) was calculated using the methods described by Karcher and Richardson (2003).

Plant color is a criterion used in plant phenotyping to assess the effect of treatment imposed during the growing period of plants and is considered as visual indicator of quality in vegetables (Barrett et al., 2010) and health in turfgrasses (Landschoot and Mancino, 2000). This is usually being done using predetermined rating scales or using color charts like Munsell Color Charts. Digital image analysis (DIA) to assess color in crops is gaining interest among researchers as an alternative approach to visual ratings to eliminate subjectivity in color phenotyping. DIA has been used in assessing color in turfgrass (Karcher and Richardson, 2003) and vegetables (Manninen et al., 2015), and even in detecting, quantifying and classifying plant diseases (Barbedo, 2013). Meanwhile, this study utilized DIA to quantify objectively the green color of artichoke seedlings grown under LED light treatments. Figure 5 shows the DGCI values and that DGCI under white light was low across genotypes (0.16) compared to natural (0.185), red (0.184), and blue (0.187) light treatments. Light treatment showed highly significant (*p* < 0.0001) influence on DGCI values. Correlation between DGCI and total chlorophyll content showed moderate positive correlation (*r* = 0.56) under white light. Other light treatments showed weak negative correlations between DGCI values and chlorophyll content. This shows that DGCI values can be used as an alternative indicator of chlorophyll content in artichoke, but protocol needs further optimization to determine if genotype could influence DGCI values. In turfgrass for instance, color evaluation under the National Turfgrass Evaluation Program, genetic color is being considered in the evaluation to cover the inherent color of the genotype being evaluated (http://www.ntep.org/reports/ratings.htm).

## Discussion

The spectral region of 400–700 nm drives photosynthetic reactions in plants (Sager and McFarlane, 1997) and the radiation in this region was defined as the photosynthetically active radiation (McCree, 1972). McCree (1972) measured action spectra on 22 crop plants to identify what regions in the visible spectrum of light activate photosynthesis. The action spectra revealed two broad maxima centered at 620 and 440 nm and a shoulder at 670 nm which correspond to the red (600–700 nm) and blue (400–499 nm) regions. The peak of the blue region is 70% of the red region that may indicate the role of red region in photosythetic activities in plants. These two maxima also correspond to the absorption spectra of chlorophyll pigments that constitute 80–95% of the lights absorbed. This provides strong evidence of the importance of blue and red regions for photosythetic activities in plants (Terashima et al., 2009). Our study showed that red light treatment enhanced the growth of artichoke seedlings. This effect of red light on plant height holds true in monocot crops as well. Rice seedlings grown under red light were taller while seedlings under blue light have inhibited shoot elongation (Chen et al., 2014).

Red light treatment also influenced root growth by as much as 71% in var. Cardoon compared to same variety grown in the greenhouse. Our findings concur with a study about grapes grown *in vitro* under red light (Poudel et al., 2008) showing high rooting percentage and root numbers compared to other light treatments. *Protea cynaroides*, a species difficult to propagate *in vitro* showed enhanced rooting of plantlets (67% rooted) when grown under red light compared to plantlets under blue or fluorescent light (13% rooted) (Wu and Lin, 2012). Although it seems unclear how light could influence root elongation when roots are covered under the soil, a study on photometric measurement of transmitted light showed that light is transmitted (0.55 μmol m^−2^s^−1^) to a depth of 8 mm in gray-white soil (Kasperbauer and Hunt, 1988). Another study reported that light-sensitive positive geotropism responses in horizontally growing maize roots were detected to a depth of 15 mm in sandy loam soil (Tester and Morris, 1987). Another avenue that light could reach the roots is through conduction of light in the stems. Studies conducted by Sun and associates (Sun et al., 2003, 2005) showed that vascular tissues in the stems and roots in 22 woody and 18 herbaceous plant species can axially conduct light. Both studies found that red and far-red regions of the light spectrum were efficiently transmitted in both tissues. In herbaceous plants, light penetrated the interior of the stem, and was conducted axially toward the roots (Sun et al., 2005). These findings indicate that light signals perceived from above-ground directly contribute to the regulation of growth and development of below-ground roots through the internal light-conducting system from stem to roots (Sun et al., 2005). Root growth is not the only physiological process influenced by red light. Afreen et al. (2006) reported that red light highly stimulated melatonin production in the roots of medicinal herb Chinese liquorice (*Glycyrrhiza uralensis*).

Light quality also affected leaf development as artichoke seedlings under red light had 28% more leaves and 26% and 39% more leaves under blue and white light, respectively when compared to greenhouse-grown artichokes. Similarly, under red light, hydroponically-grown lettuce developed more leaves than those grown under blue light (Yanagi et al., 1996) and in *in vitro* propagation of *P. cynaroides* (Wu and Lin, 2012) as well. Total shoot and root biomass followed the same trend with other plant characters measured. Compared to greenhouse-grown artichokes, above- and below-ground biomass was bulkier on artichoke seedlings grown under red light. Comparable trends were also observed in studies about Chinese cabbage and leaf lettuce. Chinese cabbage showed higher shoot and root biomass under red than under blue light conditions (Li et al., 2012). Biomass in green and red leaf lettuce under red light was three times higher than the biomass of plants grown under fluorescent light (Son and Oh, 2013) but contradicted a study that found blue light-treated lettuce showing higher biomass than those under red light treatment (Muneer et al., 2014).

The response to light is dependent upon genotype as other studies (Li et al., 2012; Muneer et al., 2014) and this study have demonstrated. Red light treatment effected increase in total chlorophyll content in rice var. Taichung (Shen 10) compared to rice seedlings grown under blue or green light treatments (Chen et al., 2014). Results of this study demonstrated that artichoke seedlings grown under white light had lower chlorophyll content and showed less biomass compared to seedlings under other light treatments. These results are expected since photosynthesis is responsible for almost all dry matter accumulation in plants (Kang and van Iersel, 2004).

## Conclusion

Light is an important factor in plant growth and development. In controlled environment agriculture, it is critical to optimize the spectral quality of the artificial light source for plant production in indoor farming facilities. The use of LED lights is becoming a popular source of artificial light for indoor farming. However, its utilization needs to be optimized because LED lights only provide a narrow spectrum of light. Using monochromatic LED lights, our study identified specific spectrum that greatly influenced the growth and development of indoor-grown artichoke seedlings. Results showed that red LED compared to natural light condition significantly influenced seedling growth and development by up to 97% increase across three artichoke genotypes. All plant characters measured exhibited 22–97% increase in red LED-treated artichoke seedlings compared to greenhouse-grown plants. Growth increase was highest in the red-light spectrum, establishing the importance of this spectrum for enhancing the growth of artichokes indoors. The study also demonstrated the contrasting effect of white and blue light treatments on seedling growth. The two light treatments resulted to 54–80% reduction in growth compared to natural-light grown plants. This study provides baseline information for indoor farming practitioners in the design of light system for indoor growing of artichokes.

## Author contributions

RR, PR, TT, and GB conceived the experiments. RR conducted the experiments. RR and TT analyzed the data. RR and PR wrote the manuscript. The manuscript was reviewed by RR, PR, TT, and GB.

### Conflict of interest statement

The authors declare that the research was conducted in the absence of any commercial or financial relationships that could be construed as a potential conflict of interest.

## Supplementary material

Supplemental Figure 1 **Comparison between LED and natural light**. Percentage difference (%) in plant response between the LED lights-grown and greenhouse-grown artichoke seedlings. Shoot biomass **(A)**, Root biomass **(B)**, Shoot/Root biomass ratio **(C)**, Plant height **(D)**, Root length **(E)** and Number of leaves **(F)**. Error bars were calculated from five biological replicates.

Supplemental Figure 2 **Comparison of LED vs. natural light across genotype**. Percentage difference of LED-grown against natural-grown seedlings. Data is the average of the three genotypes for each light treatment.

Supplemental Table 1 **Analysis of variance on the effect of light conditions on the growth of artichoke seedlings**. *p*-values in bold indicate statistical significance.

Supplemental Table 2 **Effect of variety, light conditions and their interaction on plant height (A)**, root biomass **(B)**, shoot biomass **(C)**, shoot/root ratio **(D)**, root length **(E)**, leaf number **(F)** and chlorophyll content **(G)** of artichoke. Means in column followed by the same letter are not significantly different at *p* = 0.05 based on Tukey's HSD test. GH denotes natural light.

Supplemental Table 3 **Tests for homoscedascity of residuals**.
