# Supplementary material for: Effect of Spectral Quality of Monochromatic LED Lights on the Growth of Artichoke Seedlings
Source: Front Plant Sci. 2017 Feb 17;8:190. doi: 10.3389/fpls.2017.00190 (PMC5313474; doi:10.3389/fpls.2017.00190)
Supplement: Supplemental Figure 1 — Comparison between LED and natural light. Percentage difference (%) in plant response between the LED lights-grown and greenhouse-grown artichoke seedlings. Shoot biomass (A), Root biomass (B), Shoot/Root biomass ratio (C), Plant height (D), Root length (E) and Number of leaves (F). Error bars were calculated from five biological replicates. [file Image1.pdf]

Supplemental Data

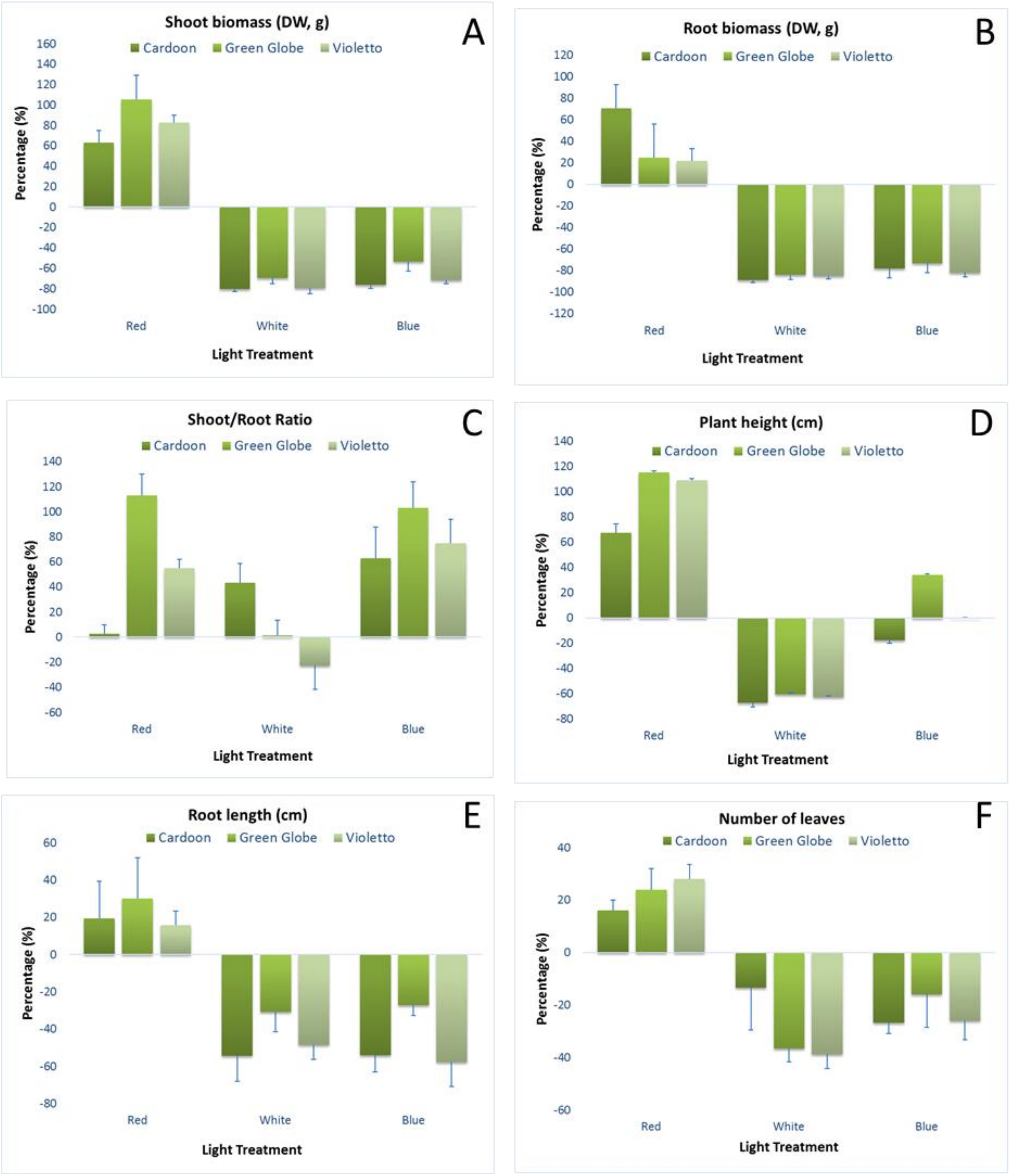

Supplemental Figure 1. **Comparison between LED and natural light**. Percentage difference (%) in plant response between the LED lights-grown and greenhouse-grown artichoke seedlings. Shoot biomass (A), Root biomass (B), Shoot/Root biomass ratio (C), Plant height (D), Root length (E) and Number of leaves (F). Error bars were calculated from five biological replicates.
