# Supplementary material for: Effect of Spectral Quality of Monochromatic LED Lights on the Growth of Artichoke Seedlings
Source: Front Plant Sci. 2017 Feb 17;8:190. doi: 10.3389/fpls.2017.00190 (PMC5313474; doi:10.3389/fpls.2017.00190)
Supplement: Supplemental Figure 2 — Comparison of LED vs. natural light across genotype. Percentage difference of LED-grown against natural-grown seedlings. Data is the average of the three genotypes for each light treatment. [file Image2.pdf]

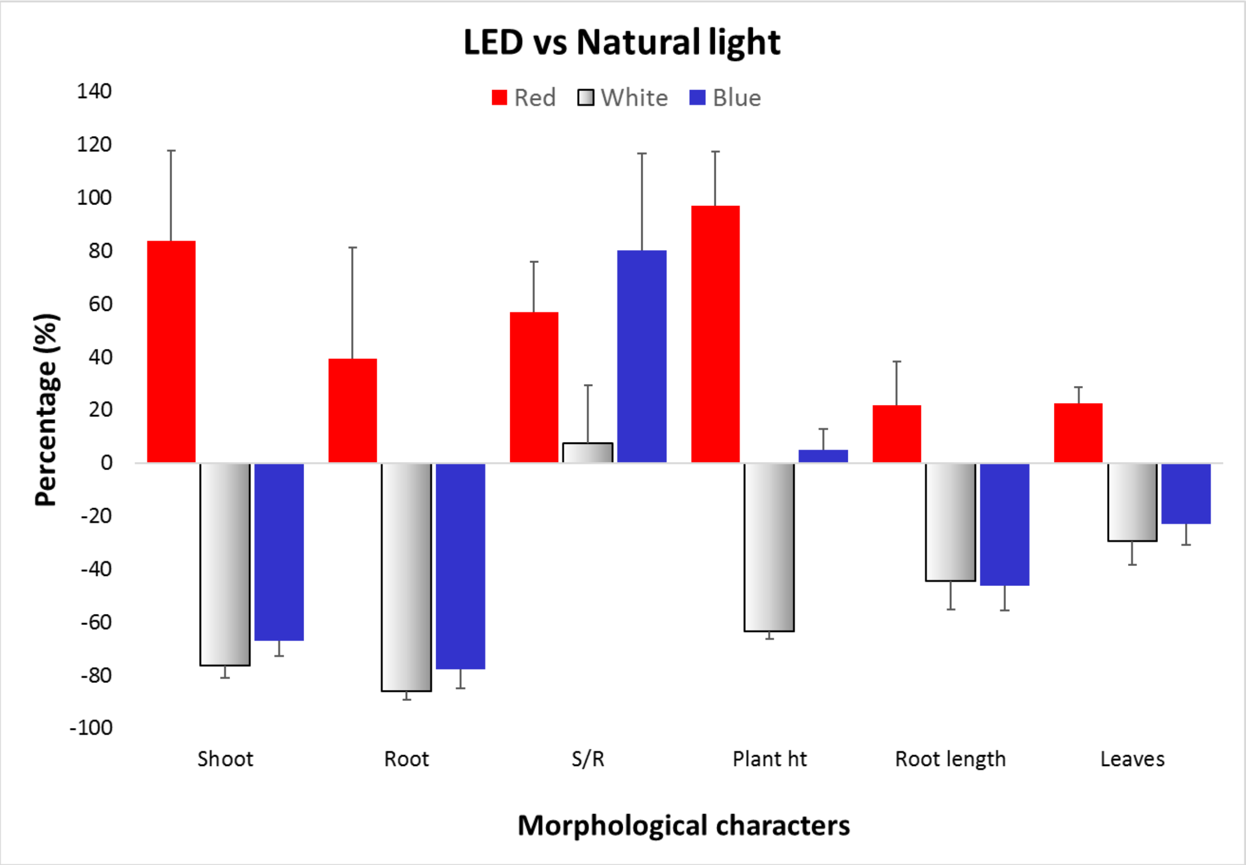

Supplemental Figure 2. **Comparison of LED vs natural light across genotype**. Percentage difference of LED-grown against natural-grown seedlings. Data is the average of the three genotypes for each light treatment.
