# Supplementary material for: Effect of Spectral Quality of Monochromatic LED Lights on the Growth of Artichoke Seedlings
Source: Front Plant Sci. 2017 Feb 17;8:190. doi: 10.3389/fpls.2017.00190 (PMC5313474; doi:10.3389/fpls.2017.00190)
Supplement: Supplemental Table 1 — Analysis of variance on the effect of light conditions on the growth of artichoke seedlings. p-values in bold indicate statistical significance. [file Table1.pdf]

Supplemental Table 1. Analysis of variance on the effect of light conditions on the growth of artichoke seedlings. P values in bold indicate statistical significance.

Source	DF	Sum of Squares	F Ratio	Prob > F
**Plant Height (cm)**				
Variety	2	25.4463	4.5807	**0.0156**
Rep	4	0.591	0.0532	0.9945
Light Type	3	1469.163	176.3129	**<.0001**
Variety*Light Type	6	51.6283	3.0979	**0.0128**
**Leaves**				
Variety	2	10.23333	7.3413	**0.0018**
Rep	4	0.933333	0.3348	0.8531
Light Type	3	38.53333	18.429	**<.0001**
Variety*Light Type	6	4.566667	1.092	0.3822
**Root length (cm)**				
Variety	2	83.0363	1.3827	0.2616
Rep	4	104.8043	0.8726	0.488
Light Type	3	2212.107	24.5575	**<.0001**
Variety*Light Type	6	212.1397	1.1775	0.3357
**Shoot Biomass (g)**				
Variety	2	0.102203	3.6014	**0.0356**
Rep	4	0.02406	0.4239	0.7905
Light Type	3	3.314547	77.8636	**<.0001**
Variety*Light Type	6	0.090743	1.0658	0.3973
**Root Biomass (g)**				
Variety	2	0.033443	9.4643	**0.0004**
Rep	4	0.0053	0.7499	0.5634
Light Type	3	0.276338	52.1348	**<.0001**
Variety*Light Type	6	0.039037	3.6824	**0.0047**
**Shoot/Root Ratio**				
Variety	2	29.02225	8.1567	**0.001**
Rep	4	14.14068	1.9871	0.1131
Light Type	3	32.78153	6.1422	**0.0014**
Variety*Light Type	6	14.2891	1.3387	0.2607
**Chlorophyll a**				
Variety	2	0.000287	0.61	0.5608
Rep	1	0.000284	1.2084	0.2951
Light Type	3	0.004848	6.8737	**0.0071**
Variety*Light Type	6	0.001384	0.9812	0.4817
**Chlorophyll b**				
Variety	2	0.005113	2.9692	0.0931
Rep	1	0.000142	0.1645	0.6928
Light Type	3	0.019485	7.5437	**0.0051**
Variety*Light Type	6	0.006452	1.249	0.3538
**Total Chlorophyll**				
Variety	2	0.007861	2.408	0.1357
Rep	1	0.000027	0.0165	0.9002
Light Type	3	0.041077	8.3885	**0.0035**
Variety*Light Type	6	0.010544	1.0766	0.4319
