# Supplementary material for: Effect of Spectral Quality of Monochromatic LED Lights on the Growth of Artichoke Seedlings
Source: Front Plant Sci. 2017 Feb 17;8:190. doi: 10.3389/fpls.2017.00190 (PMC5313474; doi:10.3389/fpls.2017.00190)
Supplement: Supplemental Table 2 — Effect of variety, light conditions and their interaction on plant height (A), root biomass (B), shoot biomass (C), shoot/root ratio (D), root length (E), leaf number (F) and chlorophyll content (G) of artichoke. Means in column followed by the same letter are not significantly different at p = 0.05 based on Tukey's HSD test. GH denotes natural light. [file Table2.pdf]

Supplemental Table 2. Effect of variety, light conditions and their interaction on plant height (A), root biomass (B), shoot biomass (C), shoot/root ratio (D), root length (E), leaf number (F) and chlorophyll content (G) of artichoke. Means in column followed by the same letter are not significantly different at *p= 0.05* based on Tukey's HSD test. GH denotes natural light.

A. Plant height (cm)

Effect of Variety on Plant Height (cm)

Variety	Least Sq Mean	
Cardoon	13.400	A
Green Globe	12.350	A B
Violetto	11.835	B

Critical q- value: 2.42548

Effect of Light on Plant Height (cm)

Light	Least Sq Mean	
Red	20.727	A
Blue	11.020	B
GH	10.927	B
White	7.440	C

Critical q- value: 2.67001

Effect of the interaction of light and variety on Plant Height (cm)

Variety*Light Type	Least Sq Mean	
Cardoon,Red	22.180	A
Violetto,Red	20.100	A
Green Globe,Red	19.900	A
Cardoon,GH	13.300	B
Green Globe,Blue	12.380	B C
Cardoon,Blue	10.920	B C D
Violetto,GH	10.060	B C D E
Violetto,Blue	9.760	B C D E
Green Globe,GH	9.420	C D E
Green Globe,White	7.700	D E
Violetto,White	7.420	D E
Cardoon,White	7.200	E

Critical q- value: 3.44911

B. Root biomass (g)

Effect of Variety on Root Biomass (g)

Variety	Least Sq Mean	
Cardoon	0.123	A
Violetto	0.083	B
Green Globe	0.067	B

Critical q- value: 2.42548

Effect of Light on Root Biomass (g)

Light	Least Sq Mean	
Red	0.174	A
GH	0.141	A
Blue	0.027	B
White	0.021	B

Critical q- value: 2.67001

Effect of the interaction of light and variety on Root Biomass (g)

Variety*Light Type	Least Sq Mean	
Cardoon,Red	0.264	A
Cardoon,GH	0.168	B
Violetto,Red	0.152	B
Violetto,GH	0.134	B
Green Globe,GH	0.122	B C
Green Globe,Red	0.106	B C D
Cardoon,Blue	0.034	C D E
Green Globe,Blue	0.026	D E
Cardoon,White	0.026	D E
Violetto,Blue	0.022	D E
Violetto,White	0.022	D E
Green Globe,White	0.014	E

Critical q- value: 3.44911

C. Shoot biomass (g)

Effect of Variety on Shoot Biomass (g)

Variety	Least Sq Mean	
Cardoon	0.372	A
Green Globe	0.301	A B
Violetto	0.274	B

Critical q- value: 2.42548

Effect of Light on Shoot Biomass (g)

Light	Least Sq Mean	
Red	0.666	A
GH	0.393	B
Blue	0.116	C
White	0.087	C

Critical q- value: 2.67001

D. Shoot/Root Ratio

Effect of Variety on Shoot/Root Ratio

Variety	Least Sq Mean	
Green Globe	5.128	A
Cardoon	3.731	B
Violetto	3.586	B

Critical q- value: 2.42548

Effect of Light on Shoot/Root Ratio

Light	Least Sq Mean	
Blue	4.959	A
White	4.370	A
Red	4.319	A
GH	2.944	B

Critical q- value: 2.67001

Supplemental Table 2. Continuation…

E. Root length (cm)

Effect of Light on Root length (cm)

Light	Least Sq Mean		
Red	27.093	A	
GH	23.420	A	
White	14.907		B
Blue	12.193		B

Critical q- value: 2.67001

F. Leaf number

Effect of Variety on Leaf number

Variety	Least Sq Mean		
Cardoon	5.050	A	
Green Globe	4.200		B
Violetto	4.150		B

Critical q- value: 2.42548

Effect of Light on Leaf number

Light	Least Sq Mean			
Red	5.667	A		
GH	4.667		B	
White	4.000		B	C
Blue	3.533			C

Critical q- value: 2.67001

G. Chlorophyll content

Effect of Light on Chlorophyll a

Light	Least Sq Mean		
Natural	0.094	A	
Red	0.087	A	
Blue	0.076	A	B
White	0.056		B

Critical q- value: 3.00951

Effect of Light on Chlorophyll b

Light	Least Sq Mean		
Red	0.100	A	
Natural	0.078	A	B
Blue	0.041		B
White	0.029		B

Critical q- value: 3.00951

Total Chlorophyll

Light	Least Sq Mean			
Red	0.189	A		
Natural	0.173	A	B	
Blue	0.119		B	C
White	0.086			C

Critical q- value: 3.00951
