# Supplementary material for: Effect of Spectral Quality of Monochromatic LED Lights on the Growth of Artichoke Seedlings
Source: Front Plant Sci. 2017 Feb 17;8:190. doi: 10.3389/fpls.2017.00190 (PMC5313474; doi:10.3389/fpls.2017.00190)
Supplement: Supplemental Table 3 — Tests for homoscedascity of residuals. [file Table3.pdf]

Supplemental Table 3. Tests for homoscedascity of residuals.

Shoot Dry weight

Test	F Ratio	DFNum	DFDen	Prob > F
**Variety**				
O'Brien[.5]	1.2632	2	57	0.2905
Brown-Forsythe	1.4727	2	57	0.2379
Levene	1.7643	2	57	0.1805
Bartlett	11.8698	2	.	<.0001
**Light**				
O'Brien[.5]	2.4651	3	56	0.0717
Brown-Forsythe	6.5935	3	56	0.0007
Levene	8.4537	3	56	0.0001
Bartlett	22.9201	3	.	<.0001
**Variety x Light**				
O'Brien[.5]	1.4317	11	48	0.1899
Brown-Forsythe	1.9669	11	48	0.0535
Levene	4.3983	11	48	0.0001
Bartlett	6.9620	11	.	<.0001

Shoot/Root Ratio

Test	F Ratio	DFNum	DFDen	Prob > F
**Variety**				
O'Brien[.5]	0.5625	2	57	0.5729
Brown-Forsythe	0.4520	2	57	0.6386
Levene	0.4856	2	57	0.6179
Bartlett	0.8346	2	.	0.4341
**Light**				
O'Brien[.5]	3.1307	3	56	0.0327
Brown-Forsythe	4.4555	3	56	0.0071
Levene	4.3263	3	56	0.0082
Bartlett	5.6597	3	.	0.0007
**Variety x Light**				
O'Brien[.5]	2.0494	11	48	0.0437
Brown-Forsythe	1.4668	11	48	0.1755
Levene	2.7378	11	48	0.0079
Bartlett	2.2790	11	.	0.0089

Root Dry weight

Test	F Ratio	DFNum	DFDen	Prob > F
**Variety**				
O'Brien[.5]	1.2184	2	57	0.3033
Brown-Forsythe	1.2131	2	57	0.3048
Levene	1.3268	2	57	0.2734
Bartlett	4.2087	2	.	0.0149
**Light**				
O'Brien[.5]	3.3147	3	56	0.0263
Brown-Forsythe	5.1949	3	56	0.0031
Levene	8.4562	3	56	0.0001
Bartlett	18.5096	3	.	<.0001
**Variety x Light**				
O'Brien[.5]	1.7517	11	48	0.0902
Brown-Forsythe	2.1219	11	48	0.0365
Levene	3.9760	11	48	0.0004
Bartlett	5.0708	11	.	<.0001

Plant height

Test	F Ratio	DFNum	DFDen	Prob > F
**Variety**				
O'Brien[.5]	0.9813	2	57	0.3811
Brown-Forsythe	1.7158	2	57	0.189
Levene	1.6487	2	57	0.2013
Bartlett	3.8126	2	.	0.0221
**Light**				
O'Brien[.5]	2.1481	3	56	0.1044
Brown-Forsythe	2.0918	3	56	0.1116
Levene	2.9842	3	56	0.0388
Bartlett	4.989	3	.	0.0018
**Variety x Light**				
O'Brien[.5]	1.3195	11	48	0.2431
Brown-Forsythe	1.1757	11	48	0.3286
Levene	2.0649	11	48	0.0420
Bartlett	2.4845	11	.	0.0041

Root length

Test	F Ratio	DFNum	DFDen	Prob > F
**Variety**				
O'Brien[.5]	2.9034	2	57	0.063
Brown-Forsythe	2.9415	2	57	0.0608
Levene	3.1496	2	57	0.0504
Bartlett	2.6107	2	.	0.0735
**Light**				
**Test**	**F Ratio**	**DFNum**	**DFDen**	**Prob > F**
O'Brien[.5]	1.8055	3	56	0.1567
Brown-Forsythe	2.3608	3	56	0.0811
Levene	3.0933	3	56	0.0341
Bartlett	1.9868	3	.	0.1135
**Variety x Light**				
**Test**	**F Ratio**	**DFNum**	**DFDen**	**Prob > F**
O'Brien[.5]	1.0885	11	48	0.3903
Brown-Forsythe	0.6865	11	48	0.7443
Levene	1.9997	11	48	0.0494
Bartlett	1.3977	11	.	0.166

DGCI

Test	F Ratio	DFNum	DFDen	Prob > F
**Variety**				
O'Brien[.5]	1.0481	2	57	0.3573
Brown-Forsythe	1.4727	2	57	0.2379
Levene	1.2087	2	57	0.3061
Bartlett	3.0618	2	.	0.0468
**Light**				
**Test**	**F Ratio**	**DFNum**	**DFDen**	**Prob > F**
O'Brien[.5]	3.7867	3	56	0.0152
Brown-Forsythe	3.8446	3	56	0.0142
Levene	3.9154	3	56	0.0131
Bartlett	6.3275	3	.	0.0003
**Variety x Light**				
**Test**	**F Ratio**	**DFNum**	**DFDen**	**Prob > F**
O'Brien[.5]	1.3762	11	48	0.2149
Brown-Forsythe	1.2076	11	48	0.3078
Levene	1.7231	11	48	0.0966
Bartlett	2.1099	11	.	0.0165
